# Pitowsky’s Kolmogorovian Models and Super-determinism

**DOI:** 10.1007/s10701-016-0049-0

**Published:** 2016-11-21

**Authors:** Jakob Kellner

**Affiliations:** Institute of Discrete Mathematics and Geometry, TU Wien, Vienna, Austria

## Abstract

In an attempt to demonstrate that local hidden variables are mathematically possible, Pitowsky constructed “spin- functions” and later “Kolmogorovian models”, which employs a nonstandard notion of probability. We describe Pitowsky’s analysis and argue (with the benefit of hindsight) that his notion of hidden variables is in fact just super-determinism (and accordingly physically not relevant). Pitowsky’s first construction uses the Continuum Hypothesis. Farah and Magidor took this as an indication that at some stage physics might give arguments for or against adopting specific new axioms of set theory. We would rather argue that it supports the opposing view, i.e., the widespread intuition “if you need a non-measurable function, it is physically irrelevant”.

## No-Go Theorems

We briefly recall the notion on hidden variables and the two no-go theorems that will be relevant in this paper: Bell’s theorem [1], the groundbreaking first proof that local hidden variables are impossible; and the Greenberger-Horne-Zeilinger (GHZ) theorem [6]. The GHZ theorem is simpler and stronger, as it shows that local hidden variables cannot be consistently assigned to a single system (of three particles); whereas Bell’s theorem shows that certain statistical frequencies cannot be reproduced by local hidden variables.

More details can be found e.g., in Mermin’s paper [12].

We will only consider systems of one, two or three spin- particles. $\sigma _{\varvec{a}}$ denotes the spin observable in direction ${\varvec{a}}$ (with possible values $\pm 1$); if we are dealing with more than one particle, the observable for the *i*-th particle is called $\sigma ^i_{\varvec{a}}$. We will only investigate idealized *Gedanken* experiments and do not care how they could be implemented in reality.

### Hidden Variables, Super-determinism

#### Local Hidden Variables

In quantum mechanics, a (pure) system is described by its state, a vector $\vert \Psi \rangle$ in some Hilbert space ${\mathcal H}$. For our purposes we can assume ${\mathcal H}$ to be finite dimensional, which somewhat simplifies notation. For a given state and an observable *A*, the result of the measurement is generally not determined; we just get probabilities for certain outcomes.

It is natural to ask whether there is a description of the system that provides deterministic predictions. Let us call such a description “hidden variables”: A system in hidden variable state *v* has predetermined results *v*(*A*) for all^1^ observables *A*. (In particular we require predictions for observables *A*, *B* which do not commute; i.e., which cannot be measured simultaneously.)

Once we perform a measurement for *A* (resulting in *v*(*A*)) then the system will change into a new hidden variable state $v'$; and if we then perform a measurement for *B* we get the result $v'(B)$. Generally there is no reason to assume that $v(B)=v'(B)$. Actually, it is obvious that for non-commuting observables, the hidden variable *has* to change: Measuring first *A*, then *B*, and then *A* again will generally result in different values for the two *A* measurements.

Let us call hidden variables *non-contextual*, if $v(B)=v'(B)$ *is* satisfied for *commuting* observables *A*, *B*; and *local*, if it is satisfied for *spatially separated* observables.^2^ In other words: if for a hidden variable state we “simultaneously” measure such *A*, *B* we get the results *v*(*A*) and *v*(*B*).

It is widely accepted that the GHZ theorem (cf. Sect. 1.2) shows that local hidden variables are impossible (assuming of course that quantum mechanical predictions are satisfied for all possible measurement combinations).

Pitowsky claims his model is even non-contextual.

#### Statistical Hidden Variables

A hidden variable model for a given quantum mechanical state $\vert \Psi \rangle$ must predict the results that are *guaranteed* by quantum mechanics.^3^ But more generally, it should also for other observables reproduce the predicted frequencies (i.e., frequencies other than 100% or 0%). So we cannot assign the same hidden variable to all systems in state $\vert \Psi \rangle$ (as there will be different results when measuring some *A* on different copies of $\vert \Psi \rangle$).

So it is natural to assume that a certain classical probabilistic mixture of different hidden variables represents $\vert \Psi \rangle$, and that we can represent a sequence of systems in state $\vert \Psi \rangle$ by “randomly” (i.e., according to the measure) picking hidden variables; and we require that the resulting frequencies are those predicted by quantum mechanics.

More formally, we can require the following:

##### Assumption 1.1

$\rho$ is a probability measure on the set of hidden variables. For all observables *A* (that we consider), $\rho (\{v:\, v(A)=a\})$ has to be equal to the quantum mechanical probability to get result *a* when measuring *A*.

(In Pitowsky’s model, a weaker notion $\rho$ will be used instead of the classical probability measure.)

It is widely accepted that Bell’s theorem (cf. Sect. 1.3) shows that local statistical hidden variables are impossible.

*Non-local* statistical hidden variables *are* possible: A very simple toy model (albeit with rather weird and unpleasant properties) is given by Kochen and Specker [8]; a more serious example is Bohm’s theory [2, 3].

#### Super-determinism

Let us define as *super-determinism* the statement:

All future phenomena are determined by the present state (not just the measurement results, but also the question which measurements are performed).

While this position might be philosophically reasonable or satisfying, it is useless for physics: It is clear that there cannot ever be a feasible “universal theory” that predicts which measurements will be performed. (We can make the measurements, i.e., the setting of some detector, depend on the arrival of photons from distance galaxies, etc.)

From a super-deterministic point of view, hidden variables are irrelevant but possible: we know which measurements will be performed, and we can (but it makes little sense to do so) assign any values we like to other measurements, and there is no reason to assume that these bogus values should satisfy any quantum mechanical prediction.

Of course we can never prove that there are no hidden variables of this “perverted” kind, as we cannot (for obvious reasons) disprove super-determinism.

Non-super-deterministic hidden variables should have the property that they predict reasonable outcomes (i.e., outcomes compatible with quantum mechanics) for *all* possible measurements (and not just for some measurements that specifically fit the hidden variable). I.e., the hidden variable should not determine or restrict which measurement we are allowed to perform.

### The Greenberger–Horne–Zeilinger (GHZ) Theorem

**Fig. 1:**
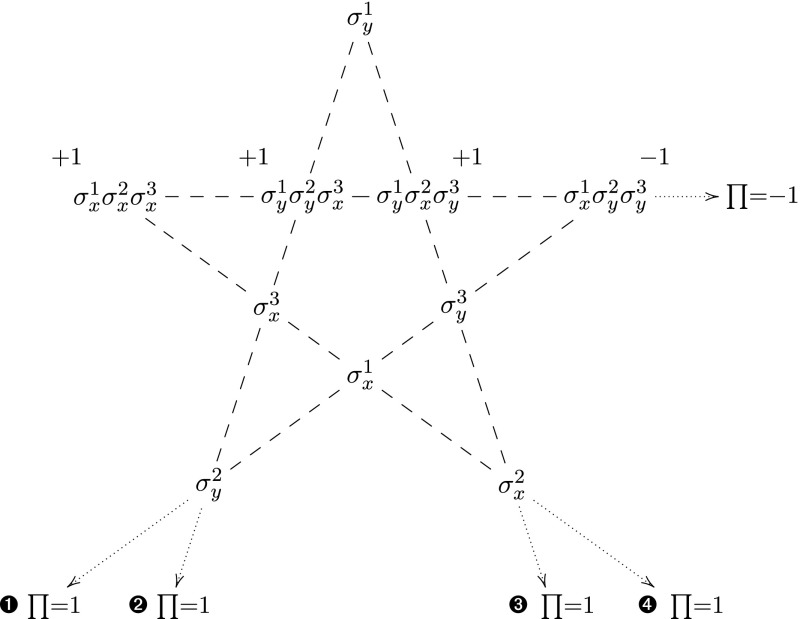
The GHZ pentagram

Consider a system of three spin- particles, and the operators listed in Fig. 1. For each of the four lines  to , the operators in the line commute and have product $+1$. Also, the four operators in the horizontal line commute and have product $-1$. We cannot assign real numbers *v*(*A*) to the operators while satisfying these five requirements.^4^ (To see this, note that each node appears in exactly two lines. So the product over the “line products”, which is $1^4\cdot (-1)=-1$, has to be the product of all *v*(*A*) squared, a contradiction.)

We now prepare the system in a simultaneous eigenstate (the so-called GHZ state) for the operators in the horizontal line, with eigenvalue $-1$ for ${\sigma ^1_x\sigma ^2_y\sigma ^3_y}$ and $+1$ for the rest. For each of the lines  to , call the product of the three remaining (single spin) operators the “reduced product”. E.g., the the reduced product of  is the product of $\sigma ^1_x$, $\sigma ^2_y$ and $\sigma ^3_y$. So for the GHZ state, quantum mechanics predicts the value $-1$ for the reduced product of , and $+1$ for –.

Also, the single spin operators are “spatially separated”, in the following sense: We can choose which line from  to  we measure by choosing directions (*x* or *y*) for each particle. For example, *xyy* results in  and *yyx* in . So if we assume that *v* is a *local* hidden variable, the result of measuring $\sigma ^2_y$ will be $v(\sigma ^2_y)$, irrespective of whether we also measure $\sigma ^1_y$ and $\sigma ^3_x$ (i.e., we measure along ) or whether we measure $\sigma ^1_x$ and $\sigma ^3_y$ (according to ).

Therefore, any local hidden variable will violate the quantum mechanically predicted value for the reduced product of at least one of the lines  to .

For later reference, let us rephrase this result using projection operators:

#### Fact 1.2

We cannot assign “yes” or ”no” to the six tests “Is $\sigma ^i_{\varvec{a}}=+1$?” such that all four of the following requirements are met (which all follow from quantum mechanics for the GHZ state):


Of course, for any given system we can only test one of the requirements  to .

### Bell’s Theorem

We now consider a pair of spin- particles in the singlet state $\frac{1}{\sqrt{2}}(\left| \uparrow \downarrow \right. \rangle +\left| \downarrow \uparrow \right. \rangle )$.

For the singlet state, the probability to get the same result for $\sigma ^1_{\varvec{i}}$ and $\sigma ^2_{\varvec{j}}$ is , where $\theta$ is the angle between $\varvec{i}$ and $\varvec{j}$. Fix three directions $\varvec{a},\varvec{b},\varvec{c}$ in the plane with angles of 120$^\circ$ between each two. So .

We now consider a hidden variable, i.e., a function *v* that maps $\sigma ^k_{\varvec{i}}$ to $v(\sigma ^k_{\varvec{i}})=\pm 1$ for $k\in \{1,2\}$ and $\varvec{i}\in \{\varvec{a},\varvec{b},\varvec{c}\}$.

If we assume that the hidden variable is local (and satisfies quantum mechanical predictions), we get

$$\begin{aligned}&v(\sigma ^2_{\varvec{i}})=-v(\sigma ^1_{\varvec{i}}).\qquad \qquad \qquad \qquad \qquad \qquad \qquad \quad {(*)} \end{aligned}$$

Not all three of the numbers $v(\sigma ^1_{\varvec{a}})$, $v(\sigma ^1_{\varvec{b}})$ and $v(\sigma ^1_{\varvec{c}})$ can be pairwise different. So if (*) holds, then there is at least one pair $\varvec{i}\ne \varvec{j}$ (call it “chosen pair”) such that $v(\sigma ^1_{\varvec{i}})\ne v(\sigma ^2_{\varvec{j}})$ (an event with quantum mechanical probability ).

Now we consider a (finite or infinite) sequence of pairs in the singlet state; and assume that the *n*-th pair is in some hidden variable state $v_n$. Assume that (*) holds (for each $\varvec{i}\in \{\varvec{a},\varvec{b},\varvec{c}\}$) with frequency at least $1-\epsilon$. So with frequency $\ge 1-3\epsilon$, (*) holds for all $\varvec{i}$ simultaneously, and then there is at at least one “chosen pair”. And as there are just three possible pairs, at least one pair $\varvec{i}\ne \varvec{j}$ has to be chosen with frequency at least  within the variables satisfying (*); i.e., with frequency at least  within all variables. When we chose $\epsilon$ to be 0.04, say, this shows:

#### Fact 1.3

It is not possible to find a (finite or infinite) sequence of hidden variables^5^ $v_n$ such that the frequency^6^ $f_{\varvec{i},\varvec{j}}$ of

1.1 $$\begin{aligned} v_n(\sigma ^1_{\varvec{i}})=v_n(\sigma ^2_{\varvec{j}}) \end{aligned}$$

is, for all $\varvec{i},\varvec{j}\in \{\varvec{a},\varvec{b},\varvec{c}\}$, within an error^7^ of at most $4\%$, equal to the quantum mechanical predicted frequencies^8^ $f^*_{\varvec{i},\varvec{j}}$ for

1.2 $$\begin{aligned} \sigma ^1_{\varvec{i}}\text { has the same result as }\sigma ^2_{\varvec{j}}. \end{aligned}$$

Assuming *local* hidden variables, (1.1) is the same as (1.2) (for each system in our sequence).

## Pitowsky’s Models

In a series of articles, Pitowsky tried to analyze whether one could escape Bell’s theorem and get *local* models by using non-measurable functions. We investigate the following articles: The first attempt [14], where he uses the Continuum Hypothesis to construct a “spin- function” and a model for the singlet state. This attempt was immediately criticised by Macdonald and, independently, Mermin [9, 11]. Starting with his response [15], Pitowsky formulated the idea of using a nonstandard notion of probability, culminating in the so-called *Kolmogorovian model* ([13] , Sect. 5] (in a construction which does not require the Continuum Hypothesis). This is a universal model that works for all quantum mechanical systems.

### Pitowsky’s First Attempt: The Singlet State

To understand Pitowsky’s analysis of Bell’s theorem, let us first give a consequence of this theorem.

#### Spin  Functions

A “spin  function” is a function $s_0$ from the sphere $S^2$ to $\pm 1$ satisfying the following:

- $$s_0(-\varvec{x})=-s_0(\varvec{x})$$.
- Fix $\varvec{x}$ and $0<\theta <\pi$ and set $S=\{\varvec{y}:\, \varvec{y}\cdot \varvec{x}=\cos (\theta ) \}$ (a circle equipped with the usual Lebesgue measure, with total measure $2\pi \sin (\theta )$). Then the set $S\cap \{\varvec{y}:\, s_0(\varvec{y})=s_0(\varvec{x})\}$ is Lebesgue measurable in *S* with relative measure .

Such a function looks promising for constructing hidden variables for the singlet state: We can define the “set of hidden variables” to be the orthogonal group $\text {O}(3)$, equipped with the normalized Haar measure $\theta$. We then define for the hidden variable $g\in \text {O}(3)$ the measurement values $g(\sigma ^1_{\varvec{a}})=s_0(g(\varvec{a}))$ and $g(\sigma ^2_{\varvec{b}})=-s_0(g(\varvec{b}))$.

If $s_0$ was additionally Lebesgue measurable, then these hidden variables would actually work and violate Bell’s theorem. So there cannot be a Lebesgue measurable spin  function.^9^ However, Piowsky shows in [14]:

##### Theorem 2.1

Assuming the Continuum Hypothesis, there is a (non measurable) spin- function.

Pitowsky then claims that the local hidden variables for the singlet state (defined as above), work and do not suffer from Bell’s theorem.

It is not entirely clear to us from the paper [14] how he thought that this would actually work out. Apparently he assumed that by “sabotaging” Bell’s proof (by using non-measurable functions), and maybe using an intuition similar to the one outlined in footnote 9, the problem would go away.

#### Criticism

But of course it does not. This was pointed out immediately [9, 11]:

Let us paraphrase the criticism in concrete terms (we use the notation of Sect. 1.3): Let us fix for example $N=50\,000$, and generate *N* many singlet states. Quantum mechanics tells us that for each pair $\varvec{i},\varvec{j}$ from $\{\varvec{a},\varvec{b},\varvec{c}\}$ the frequency of the event “$\sigma ^1_{\varvec{i}}\text { equals } \sigma ^2_{\varvec{j}}$” will (virtually certainly) be very close to the calculated probability of . More concretely, with probability $>1-10^{-91}$ (i.e., “always”) the frequency will be greater than . But Bell’s theorem shows that *any* sequence of hidden variables, *irrespective of whether they were created by a classical random process, or some nonstandard method* either gives a frequency $<f_0$ for at least one such pair $\varvec{i},\varvec{j}$, or gives a frequency of $>0.04$ to the (quantum mechanically outright impossible) event “$\sigma ^1_{\varvec{i}}\text { equals } \sigma ^2_{\varvec{i}}$” for some $\varvec{i}$.

#### Response, Towards the Universal Model

In response [15], Pitowsky indicated that the objections are based on the “classical” notion of probability, which has to be modified. We need a new notion of probability (let us call it Pitowsky probability). In this notion, it can happen that two sets (or: events) *A*, *B* have Pitowsky probability one, but the intersection $A\cap B$ has “classical” probability zero.

Actually, it turns out that the intersection even has to have small Pitowsky probability, as [16, p. 164] elaborates: According to his new notion of probability, it is no problem if “90% all of the objects are red” and “90% all of the objects are small” but “no object is small and red”.^10^

Note that Pitowsky’s spin  model features different notions of probability and certainty: By design, $\sigma ^1_{\varvec{a}}=-\sigma ^2_{\varvec{a}}$ always holds. Given $\sigma ^1_{\varvec{a}}=+1$, the correct relative frequency for for $\sigma ^1_{\varvec{b}}=+1$ is provided by classical probability (i.e., Lebesgue measure). However, the absolute frequency for $\sigma ^1_{\varvec{a}}=+1$ cannot be provided by classical probability (as there is no measurable spin  function), instead Pitowsky evokes his new notion of probability.

It turns out that once you agree to use Pitowsky probabilities, and abandon the use of classical Lebesgue measure for the relative frequencies as well, you get a much simpler model, and furthermore a “universal” model (called Kolmogorovian model by Pitowsky), that applies not only to the singlet state, but to any quantum mechanical situation. This is what Pitowsy does in [13, Sect. 5]. The new construction does not require the Continuum Hypothesis anymore (but Pitowsky uses the Axiom of Choice).

In this sense (also in Pitowsky’s presentation) the Kolmogorovian model replaces the spin  function model for the singlet state, so we will concentrate on the Kolmogorovian model in the following.

### Pitowsky’s Universal Kolmogorovian Model

In this section, we will present Pitowsky’s [13, Sect. 5] “universal” Kolmogorovian model. This model deals with (orthogonal) projection operators only. I.e., instead of predicting which real numbers will be the result of a measurement, we just predict whether a test will result in “yes”.^11^

#### Kolmogorovian Models

Let us give us Pitowsky’s definition of a mixture and a Kolmogorovian model right away: Fix a $\sigma$-algebra $\Sigma$ of (not necessarily Lebesgue measurable) subsets of [0, 1].

- A mixture $\rho :\Sigma \rightarrow \mathbb {R}$ is a monotone function such that $\rho (X)$ is between the inner and the outer Lebesgue measure of *X*.^12^
- A Kolmogorovian model consists of the following objects, with the following properties:
  I [0, 1] is the set of hidden variables. A hidden variable $\lambda \in [0,1]$ maps each projection *A* to some $\lambda (A)\in \{0,1\}$ (the result of *A* when measuring the system in the hidden variable state $\lambda$). We set $X_A:=\{\lambda \in \Omega :\, \lambda (A)=1\}$; and require that $X_A\in \Sigma$.
  II Given (a specific method to prepare) a quantum mechanical state $\vert \Psi \rangle$, we fix a mixture $\rho$ (which tells us how the hidden variable states of the state $\Psi$ are distributed; i.e., how likely a specific variable $\lambda$ will occur).
  III If *A* and *B* can be measured simultaneously (i.e., they commute), then
    $$\begin{aligned} \rho (X_A\cap X_B)+\rho (X_A\cup X_B)=\rho (X_A)+\rho (X_B). \end{aligned}$$
  IV In a “random sample” of copies of the state $\vert \Psi \rangle$, the relative frequencies of the outcome 1 for *A* (i.e., for $\lambda \in X_A$) “approaches” $\rho (X_A)$ as the sample grows.

Contrast this definition with the “statistical hidden variables” of Sect. 1.1: Instead of using a “classical” probability measure, $\rho$ is now just a “mixture”. Also, for a measure the item corresponding to IV is trivially satisfied (it is just a version of the law of large numbers); while now IV is not even well defined^13^ (it is not clear what either “random” or “approaches” actually mean; as we will see later “approaches” cannot mean that a limit in the usual mathematical sense exists).

#### Generalized Kolmogorovian Models

Instead of directly presenting Pitowsky’s construction of a Kolmogorovian model, we will first give a simpler (but really equivalent) construction for a modified notion.

Let us first note that it is physically irrelevant to require the hidden variables to be real numbers (as opposed to, say, vectors in a Hilbert space); and to require that $\rho (X_A)$ should have anything to do with inner or outer Lebesgue measures. Dropping these artificial requirements, we get:

##### Definition 2.2

Fix an arbitrary set $\Omega$ (the set of hidden variables) and $\Sigma$, a $\sigma$-algebra of subsets of $\Omega$.

- A **generalized mixture** $\rho$ is a monotone function from $\Sigma$ to $\mathbb {R}$.
- A **generalized Kolmogorovian model** is a Kolmogorovian model that uses $\Omega$ instead of [0, 1] as set of hidden variables and a generalized mixture instead of a mixture.^14^

We can now (without using the Axiom of Choice or non-measurable sets) construct a trivial (and, of course, physically useless) generalized Kolmogorovian model: We fix a quantum mechanical system with Hilbertspace ${\mathcal H}$, and a state $\vert \Psi \rangle$.

- We let $\Omega$ be the (pure) quantum mechanical states (i.e., elements^15^ of ${\mathcal H}$ with norm 1).
- For a hidden variable $\lambda \in \Omega$ and an (orthogonal) projection *A* (which we identify with its image $H_A$), we set $\lambda (A)=1$ if $\lambda$ is eigenvector of *A* with eigenvalue 1, i.e., if $\lambda \in H_A$. (And we set $\lambda (A)=0$ otherwise.) Accordingly $X_A=\{\lambda :\, \lambda (A)=1\}$, which is the set of normalized elements of $H_A$.
- We let $\Sigma$ be the set of all subsets of $\Omega$.^16^
- For $Y\in \Sigma$, let $\langle Y\rangle$ be the ${\mathcal H}$-subspace generated by *Y*.^17^ We set $\rho (Y)$ to be the quantum mechanical probability (using the state $\vert \Psi \rangle$) for the orthogonal projection to the subspace $\langle Y\rangle$ resulting in $+1$. So $\rho (X_A)$ is the quantum mechanical probability for *A* resulting in $+1$.

It is easy to check that $\Omega ,\Sigma ,\rho$ forms a generalized Kolmogorovian model:

$\rho$ is a generalized mixture as it is monotone. I and II are obvious. III is satisfied as well: Assume *A* and *B* commute. Then $\rho (X_A)$, $\rho (X_B)$, $\rho (X_A\cap X_B)$ and $\rho (X_A\cup X_B)$ are the quantum-mechanically predicted vales for a positive outcome of *A*, *B*, “*A* and *B*”, “*A* or *B*”, respectively; and therefore satisfy the equality in III.^18^

##### Remark

Other than $X_A\cap X_B$, $X_A\cup X_B$ is generally not the set of unit vectors of a subspace of ${\mathcal H}$.

#### From Generalized Kolmogorovian Models to Regular Ones

We now use $\Omega ,\Sigma ,\rho$ to recover Pitowsky’s construction of a Kolmogorovian model $\Sigma ',\rho '$ which satisfies the original definition (which, as we would like to stress again, has the same physical contents as the generalized notion). For this, we just add a bit of simple measure theory:

Fist note that the set $\Omega$ of states has size continuum. Write the interval [0, 1] as the disjoint union of continuum many sets $(Y_i)_{i\in \Omega }$ of outer Lebesgue measure 1. (Here we use the Axiom of Choice.)

We now declare a real $r\in [0,1]$ to be a hidden variable. Such an *r* is element of exactly one $Y_\lambda$ for $\lambda \in \Omega$; and we let *r* produce the same predictions as $\lambda$ in our generalized Kolmogorovian model $r(A)=\lambda (A)$.

In particular, $X'_A=\{r\in [0,1]:\, r(A)=1\}=\bigcup _{\lambda \in X_A}Y_\lambda$ will contain some of the “blocks” $Y_\lambda$ completely, and will omit others completely, more formally:

* $$\begin{aligned} \text {If} \ r\in X'_A \ \text {and}\ s \ \text {is in the same block}\ Y_\lambda \ \text {as}\ r, \ \text {then}\ s\in X'_A. \end{aligned}$$

So we really just replace a single vector $\lambda \in \Omega$ with the block $Y_\lambda$.

Let $\Sigma '$ be the $\sigma$-algebra generated by the sets $X'_A$. Then every element $X'$ of $\Sigma$ satisfies (*) as well.^19^ In particular, whenever $X'\in \Sigma '$ is neither empty nor equal to [0, 1], it has outer measure 1 and inner measure 0 (cf. the end of Footnote 12).

For $X'\in \Sigma$, we set $X=\{\lambda \in \Omega :\, Y_\lambda \subseteq X'\}$ and $\rho '(X')=\rho (X)$. In particular, $\rho '(X'_A)=\rho (X_A)$, which is the quantum mechanical probability (assuming state $\vert {\Psi }\rangle$) for *A* resulting in $+1$.

$\Sigma ',\rho '$ is a Kolmogorovian model:

As $\rho$ is monotone, so is $\rho '$. We have seen $0=m_*(X')\le \rho '(X')\le m^*(X')=1$ for nontrivial $X'\in \Sigma$, and for the trivial cases note that $\rho '(\emptyset )=0$ and $\rho '([0,1])=1$. So $\rho '$ is a mixture. I and II are obvious. For III, it is enough to note that $X'_A\cup X'_B$ consists of the blocks $Y_\lambda$ that satisfy $\lambda \in X_A\cup X_B$, and so $\rho '(X'_A\cup X'_B)=\rho (X_A\cup X_B)$. The same holds for $\cap$ instead of $\cup$. So the required equation in III for $\rho '$ follows from the equation for $\rho$.

#### A Simple Example

Already a very simple example shows the strange nature of Pitowsky’s model:^20^

Let us look at a pair of spin- particles in the singlet state. We use the “generalized” notation (a hidden variable is a state $\phi$ in the four dimensional Hilbert space), which can trivially be translated into Pitowsky’s original notation (then the hidden variable is a real *r* that gets mapped to $\phi$).

The hidden variable $\phi$ will result in $\sigma ^1_{\varvec{a}}=+1$ if and only if $\phi =\left| \varvec{a} \right. \rangle \otimes v$ for some $v\in {\mathcal H}_2$ (the two-dimensional space of the second particle); and will result in $\sigma ^2_{\varvec{a}}=+1$ if and only if $\phi =v\otimes \left| \varvec{a} \right. \rangle$ for some $v\in {\mathcal H}_1$.

Measuring $\sigma ^1_{\varvec{a}}$ will not interfere with the $\sigma ^2_{\varvec{a}}$ hidden-variable measurement, as the hidden variable model is local.^21^

Let us denote with $X_{\varvec{a}}$ the set of hidden variables that have the form either $v\otimes \left| \varvec{a} \right. \rangle$ or $\left| \varvec{a} \right. \rangle \otimes v$. As we can perform both (spatially separated) measurements, and as the Pitowsky-probabilities correspond to the quantum mechanically predicted probabilities, we know that $X_{\varvec{a}}$ has Pitowsky-probability 1 (i.e., $\rho (X_{\varvec{a}})=1$).

So for three different directions $\varvec{a}$, $\varvec{b}$ and $\varvec{c}$ the sets $X_{\varvec{a}}\cap X_{\varvec{b}}$ have at most two elements while

2.1 $$\begin{aligned} X_{\varvec{a}}\cap X_{\varvec{b}}\cap X_{\varvec{c}}\text { is empty.} \end{aligned}$$

(Really empty, not just of probability 0.)

So, to paraphrase Pitowsky: *All* balls are red, and *all* balls are small, and *all* balls are heavy, but *no* ball is small and red and heavy.

While this effect is most obvious in the Kolmogorovian model, similar effects apply to the earlier model for the singlet state^22^ due to Bell’s theorem, as described in sections Criticism and Response on page 5 and also in [7, p. 1331]; see also Czachor’s paper [4].

## Analysis of the Kolmogorovian Model

### Kolmogorovian models are superdeterministic

#### The Weirdness of Quantum Mechanics

To work with another example,^23^ let us move from the singlet state to the GHZ state. According to Fact 1.2, quantum physics implies that each of – is satisfied, and that this cannot be done by fixing hidden parameter values $\pm 1$ for the six $\sigma ^i_{\varvec{a}}$ (for $i\in \{1,2,3\}$ and $\varvec{a}\in \{x,y\}$).

So all deterministic hidden variables theories have to be “weird” in some way or the other. They have to suffer from one of the following:

- Nonlocality. As mentioned, there are such non-local hidden variable models.
- Super-determinism. As mentioned, physically doubly irrelevant: Firstly super-determinism is physically unfeasible, secondly hidden variables are pointless within super-determinism.
- Non-classical logic. One could maybe claim that – *can* hold simultaneously for suitable “yes/no” values, if we completely change our basic understanding of logic and reasoning. We will ignore such positions here.

#### Pitowsky’s Probability

Pitowsky however claims to have found another way: supposedly one can move all the weirdness into his nonstandard notion of *statistics* (Pitowsky probability). He explicitly claims [13, p. 169] that his model is local (even non-contextual) and classical, i.e., does not suffer weirdness (a) or (c). We will argue in the following that Pitowsky’s model is in fact just super-deterministic.

Let $A_1$ be the set of hidden Kolmogorovian variables satisfying . As quantum mechanics implies , $\rho (A_1)=1$. In other words, the Pitowsky probability for a hidden variable to be in the set $A_1$ is 1. Analogously define $A_2$, $A_3$ and $A_4$. So $A_i$ all are Pitowsky measure 1 sets; while the intersection $\bigcap _{i=1,\dots ,4}A_i=\emptyset$ is empty.

For Pitowsky, this is a peculiar property of his notion of probability:  is satisfied with measure 1 (i.e., always) as $A_i$ has measure 1; the same holds for  etc. It is true that no hidden variable can have all properties –, but if we measure property  on some sequence, then  will be satisfied; and  has to be measured on another sequence so consistently we can assume that on this sequence  is satisfied, etc.

While Pitowsky claims that this can be seen as an effect of a non-classical notion of probability, it really is just a variant of super-determinism. This can be seen most clearly if we just consider a single state three-particle system, which we *first* assume to have hidden variable *v*. We know that *v* determines the six values used in the requirements –, and therefore we know that at least one of the requirements fail. Let us assume that  fails. In other words: If we test , we will get a contradiction to quantum mechanics. However,  will hold with Pitowsky probability 1, i.e., always. So in the Pitowsky model, we *will not* measure , i.e., our hidden variable excludes certain measurements, i.e., is super-deterministic.

#### Isn’t A Single System Unrealistic?

One could object to the use of a single three-particle system: In real world experiments, one has to deal with non-perfect measurements etc., and a violation of quantum mechanic predictions in a single event would just be considered an outlier and ignored, quite consistently with experimental data.

Of course, this argument does not help: Assume that we *first* create a sequence of a million hidden variables. Each of the variables will violate at least one of the requirements –; so at least one requirement has to be violated in at least 25% of the variables. Again, assume that is the case for . If the Pitowsky setup were non super-deterministic, we could now choose to test  on the sequence, and get a success rate of at most 75% (instead of the 100% predicted by quantum mechanics); which is definitely not in line with real life experiments.

Two remarks:

- If each time we choose the one forbidden setting, we of course even get 100% failure. But it seems hard to argue that we could guess a long sequence of forbidden settings correctly.
- The argument relies on locality only; it is not required that the choice of a measurement at position 1, say, will not affect the outcomes for subsequent measurements at the same position 1. (However, in Pitowsky’s Model the outcome is in fact independent). So we generally have to assume that the result for, e.g., the *n*-th measurement of $\sigma ^1_x$ also depends on the measurement setting (*x*, *y* or *z*) of all the previous $n-1$ many measurements at position 1 (and the measurement results, but they are determined anyway). This does not change the argument: Using only locality, we can still argue that the hidden variables will give for given *n*, *i*, and $\varvec{a}$ the same results for testing $\sigma ^i_{\varvec{a}}$ on the *n*-th particle for both appearances in the equations –.

#### The Singlet State

In the GHZ case, we have seen that at least one out of four measurement combinations will be ruled out by the hidden variable. In the singlet state, super-deterministic nature of the Kolmogorovian model is even more dramatic.

Let us *first* create a hidden variable state. Using the notation of (2.1), this state can be in $X_{\varvec{b}}$ (the states that are “OK” for $\varvec{b}$) for at most two directions, $\varvec{b}_1$ and $\varvec{b}_2$, say. So whenever we *afterwards* chose to measure $\sigma ^1_{\varvec{c}}$ and $\sigma ^2_{\varvec{c}}$ for *any* direction $\varvec{c}$ other than $\varvec{b}_1$ and $\varvec{b}_2$, we get “not $+1$” both times, in a violation of quantum mechanics.

So if we have a device that can measure spins in $\ell$ many directions, and if we use two of these devices, spatially separated, and make sure both will measure the same direction,^24^ then for any given hidden variable state, at least $\ell -2$ out of the $\ell$ possible measurements will violate quantum mechanics.

### From Super-determinism to the Kolmogorovian Model, Pitowsky’s Law of Large Numbers

We have argued that Kolmogorovian models imply a form of super-determinism.

In turn, from a super-deterministic point of view, we can easily see how the Kolmogorovian model works. This will also shed light on how to interpret the notions in assumption IV of the definition of the Kolmogorovian model:

“In a random sample of particles, whose statistical state is given by the mixture $\rho$, the frequency of particles having property *A* approaches $\rho (A)$ as the sample grows.” [13, p. 163]

Recall that $\rho (A)$ is just the quantum mechanical probability for *A*.

So let us assume a super-deterministic position. We know (since we know all) that we will investigate a sequence of *m* many systems (in state $\vert \Psi \rangle$), and that for each copy of the system we will measure the sequence $A_1,A_2,\dots ,A_n$ of commuting^25^ projections. We know that the *j*-th system will give the results $a^j_1,\dots ,a^j_n$. If *m* is large enough, then (with high probability) the $a^j_i$ will appear with frequencies close to the quantum mechanically predicted sequences. For each *j*, there is a state $\phi ^j$ which is simultaneous eigenvalue of $A_i$ with eigenvalue $a^j_i$. This is the *j*-th hidden variable we chose.

So in this construction, where we start from super-determinism, the notions in assumption IV are the following: “random” means according to our prescience (i.e., quite determined and not random at all). “Approaches” means the usual limit, but only for those *A* that are actually measured. (Other observables *A* will feature frequencies entirely different from $\rho (A)$.)^26^

In this sense, Pitowsky’s model is logically consistent (just as super-determinism is). Of course, this is not particularly satisfying; but as we have argued over and over, we cannot do better.

Let us argue the same thing once again, from a slightly different point of view:

Classically, we have the following (which can be seen as a simple form of the law of large numbers):

Fix finitely (or countably) many Lebesgue measurable sets $A_i\subseteq [0,1]$ of measure $a_i$. Generate a random^27^ sequence $(r_n)_{n\in \mathbb N}$ of elements of [0, 1]. Then with probability 1, the frequency of $r_n\in A_i$ is $a_i$ for all *i*.^28^

Pitowsky observes that he cannot prove a similar law for his notion of probability; and replaces it with Principle IV in his definition of Kolmogorovian models, quoted above.

It is true that a “proper” Pitowsky law of large numbers would be sufficient to give a realistic, local, non-super-deterministic hidden variable theory; but such speculation is vacuous as it is easy to see that the “proper” law fails for Pitowsky probability. Let us again use the GHZ notation:

Fix sets $A_i\subset [0,1]$ ($i\in \{1,2,3,4\}$) such that $\bigcap A_i=\emptyset$. They do not have to be Lebesgue measurable (but we will assume they are “Pitowsky measurable” and all have “Pitowsky measure” 1). Generate (by whatever process you like, e.g., a Pitowsky random process) an infinite sequence $(r_n)_{n\in \mathbb N}$ of elements of [0, 1]. Then for at least one $i\in \{1,2,3,4\}$, the frequency of the property $r_n\in A_i$ is not 1.

Actually, if all frequencies exist^29^ then for at least one *i* the frequency is ${\le }$0.75.

The proof is entirely trivial: Fix any sequence $r_n$, and assume that all frequencies are defined and bigger than 0.75. I.e., for each $i\in \{1,2,3,4\}$ there is a $N_i$ such that for all $n\ge N_i$ the $A_i$-frequency of the finite sequence $r_1,\dots ,r_n$ is bigger than 0.75. Let *n* be $\max (N_1,N_2,N_3,N_4)$. Then the finite sequence up to *N* has frequency bigger than 0.75 for all *i*, a contradiction of the fact that $\bigcap A_i=\emptyset$.

Note that our observations in this section do not contradict anything Pitowsky says directly. Rather, Pitowsky argues that we measure the different $A_i$ on different samples, and that on each sample we will get the correct frequency for the according measurement. (As already mentioned, this is logically consistent, as it just means that we are dealing with super-determinism.)

### Set Theory and Physics

There has been some debate over the following question:

Could the choice of set theoretic axioms have any effect on the physical theories? Or, from a different perspective: could physical knowledge imply that specific set theoretic axioms should be preferred over others?

Of course we know that, e.g., the Axiom of Choice is required for many mathematical theorems (such as: every vector space has a basis), which in turn can be applied in physics. However, on closer inspection it turns out that for all concrete instances that are used, the axiom of choice is not required. The same applies even for the existence of an infinite set: One can use a very constructive, “finitary” form of mathematics that is perfectly sufficient for physics. (It is a different question whether it is equally practical and intuitive as the “usual” mathematics based on set theory.)

Pitowsky used the Continuum Hypothesis to construct a spin--function model. Pitowsky suggested that the existence of such a function might not follow from the usual axioms of set theory alone, which has recently been confirmed by Farah and Magidor [5]. In the same paper, as well as in [10], it has been argued that the spin- model is an indication that physical considerations might provide input on which new axioms should be adopted for set theory.

We do not share this opinion:

- Pitowsky’s spin--function is introduced just to “sabotage” a specific argument in one of the possible proofs of Bell’s theorem: Using a non-measurable function prevents proofs using the expected value of the function. Accordingly, the spin--function alone does not give any model; the real point of the matter is the dramatically altered basic notion of probability which has to be added on top of it (let us call it Pitowsky probability).
- Once you adopt Pitowsky probability, the spin--function becomes unnecessary: It is easy to give a universal model for all quantum mechanical systems (the Kolmogorovian model). Pitowsky uses the Axiom of Choice for this model, but we think even that is unnecessary^30^, as argued in the section on generalized Kolmogorovian models in Sect. 2.2.
- But in the end, Pitowsky probability turns out to be just a variant of super-determinism. Accordingly, the models are obviously consistent, but physically not relevant. (And doubly so: super-determinism is physically unfeasible, and hidden variables are pointless within super-determinism.)

So we come to quite the opposite conclusion as Farah and Magidor: Instead of indicating connections between physics and set theory, Pitowsky’s attempts of hidden variables rather seems to reaffirm the old intuition: “if nontrivial set theory, non-constructive mathematics or a non-measurable set is used in an essential way, it cannot be physically relevant”.

## Footnotes

1 Or just: sufficiently many; finitely many are enough for our purposes.

2 Note that non-contextual implies local, as spatially separated observables have to commute (to prevent superluminal communication).

3 I.e.: If $\vert \Psi \rangle$ is eigenvector of *A* with eigenvalue *a*, the we require $v(A)=a$. Note that the GHZ theorem shows that not even that can be done with local hidden variables.

4 The “requirement corresponding to ” is $v(\sigma ^1_y)\cdot v(\sigma ^1_y\sigma ^2_y\sigma ^3_x)\cdot v(\sigma ^3_x)\cdot v(\sigma ^2_y)=1$, etc.

5 Here, $v_n$ can be any assignment from the set $\{\sigma ^k_{\varvec{i}}:\, k=1,2,\ \varvec{i}=\varvec{a},\varvec{b},\varvec{c} \}$ to $\pm 1$.

6 In case of infinite sequences we might require the frequency (i.e., the limit) to exist.

7 I.e., $$\left| {f_{\varvec{i},\varvec{j}}-f^*_{\varvec{i},\varvec{j}}}\right| <0.04$$.

8 which are  for $\varvec{i}\ne \varvec{j}$ and 0 for $\varvec{i}=\varvec{j}$.

9 In more detail: Assume towards a contradiction that $s_0$ is a Lebesgue measurable spin  function. We now calculate the probability $p_{\varvec{i},\varvec{j}}$ for the event $g(\sigma ^1_{\varvec{i}})=g(\sigma ^2_{\varvec{j}})$, i.e., for $s_0(g(\varvec{i}))\ne s_0(g(\varvec{j}))$, i.e. for the two measurements having different outcome. A “random” choice of *g* corresponds to first “randomly” choosing a direction $\varvec{i}'$ (which is $g(\varvec{i})$), and then, again “randomly”, another direction $\varvec{j}'$ such that $\varvec{i}'$ and $\varvec{j}'$ have the same angle $\theta$ as $\varvec{i}$ and $\varvec{j}$ (this $\varvec{j}$ is $g(\varvec{j})$; note that *g* preserves angles). The probability that $s_0(\varvec{j}')=s_0(\varvec{i}')$ is, by the assumption, , so $p_{\varvec{a},\varvec{b}}$ is  as predicted by quantum mechanics. (Formally, we have to calculate $\theta (X)$, the $\text {O}(3)$ Haar measure of the set $X=\{g\in \text {O}(3):\, s_0(g(\varvec{a}))\ne s_0(g(\varvec{b}))\}$.) So we can, e.g., pick randomly an infinite (or: sufficiently large) sequence of hidden variables. By the law of large numbers, with probability one (or: sufficiently large, nonzero is enough) we will get the correct frequencies (or: frequencies that are sufficiently close), contradicting Bell’s theorem.

10 We will later even see the following form of the effect: “All objects are red” and “all objects are small” but “no object is small and red”. This form was pointed out by [7, p. 1331], who quite poignantly described the “paradoxical nonphysical nature” of the model and clarified some misunderstanding surrounding it. We have not found Pitowsky explicitly acknowledging this form, but it is part of his treatment of the Bell-KS Theorem in [13, 16].

11 When investigating which kind of hidden variables might exist, the restriction to projections does not make a substantial difference.

12 Not every $X\subseteq [0,1]$ is (Lebesgue) measurable (at least assuming the Axiom of Choice); but for every *X* there is the inner measure $m_*(X)$ and the outer measure $m^*(X)$ with the following property: $m_*(X)$ is the maximal measure of any measurable subsets $Y_*$ of *X*; and $m^*(X)$ is the minimal measure of any measurable $Y^*$ containing *X*, i.e., $Y^*\supseteq X$. Of course $m_*(X)\le m^*(X)$, and equality holds if and only if *X* is measurable (and then the measure of *X* is $m_*(X)= m^*(X)$). If $X_1$ and $X_2$ are disjoint, i.e., $X_1\cap X_2=\emptyset$, and $X_1$ has outer measure 1, then $X_2$ has inner measure 0. To see this, note that any $Y_*\subseteq X_2$ satisfies $[0,1]\setminus Y_*\supseteq X_1$, so if $Y_*$ had measure $\epsilon >0$ then $[0,1]\setminus Y_*$ had measure $1-\epsilon <1$.

13 Pitowsky mentions that requirement IV is “not a mathematical theorem, it is merely a consistent claim” [13, p. 163]. We would not even call it a claim, as the notions are undefined.

14 More verbosely, the definition is identical to the definition of Kolmogorovian model, where in Item I we replace [0, 1] with $\Omega$ and in Item II “mixture” with “generalized mixture”.

15 We could instead use one-dimensional subspaces, but that would make the notation slightly less convenient.

16 Or we could let $\Sigma$ be the $\sigma$-algebra generated by the sets $X_A$, it doesn’t matter.

17 In the infinite dimensional case we might want to use “closed subspace” instead of “subspace”.

18 In more detail: Fix an orthonormal basis ${\mathcal L}$ of common eigenvectors for *A* and *B*. Then ${\mathcal L}$ is the disjoint union of ${\mathcal L}_i$ for $i={A\cap B},{A\setminus B},{B\setminus A}, \lnot (A\cup B)$, where ${\mathcal L}_{A\cap B}$ consists of 1-eigenvectors for both *A* and *B*, ${\mathcal L}_{A\setminus B}$ consists of vectors that are 1-eigenvectors for *A* and 0-eigenvectors for *B*, etc. Then $\langle X_A\cap X_B\rangle = \langle {\mathcal L}_{A\cap B}\rangle$, and thus $\rho (X_A\cap X_B)$ is “the probability for $\langle {\mathcal L}_{A\cap B}\rangle$”, i.e., $\Sigma _{v\in {\mathcal L}_{A\cap B}}{\left| {\left\langle \Psi \phantom {v} \right| \left. \right. \rangle }\right| }^2$. Similarly, $\langle X_A\rangle =X_A=\langle {\mathcal L}_{A\cap B}\cup {\mathcal L}_{A\setminus B}\rangle$, so $\rho (X_A)=\Sigma _{v\in {\mathcal L}_{A\cap B}\cup {\mathcal L}_{A\setminus B}} {\left| {\left\langle \Psi \phantom {v} \right| \left. \right. \rangle }\right| }^2$. Analogously for $X_B$. And $\langle X_A\cup X_B\rangle =\langle {\mathcal L}_{A\cap B}\cup {\mathcal L}_{A\setminus B}\cup {\mathcal L}_{B\setminus A}\rangle$. So $\rho (X_A\cup X_B)=\Sigma _{v\in {\mathcal L}_{A\cap B}\cup {\mathcal L}_{A\setminus B}\cup {\mathcal L}_{B\setminus A}} {\left| {\left\langle \Psi \phantom {v} \right| \left. \right. \rangle }\right| }^2$.

19
$\Sigma$ could be defined as closure under arbitrary unions instead of just countable ones, or even as the set of all subsets of [0, 1]. In the latter case we just have to redefine $\rho '(X')$ as $\min \left( m_*(X'),\max \left( m^*(X'),\rho (X)\right) \right)$.

20 An even simpler example for the same effect uses just a single spin- particle; where we have to use non-contextuality instead of locality.

21 As explicitly claimed in [13, (b) on p. 169].

22 This specific example however does not work there, as $\sigma ^1_{\varvec{a}}=-\sigma ^2_{\varvec{a}}$ always holds.

23 In [13] Pitowsky does not mention the GHZ Theorem, which was not yet known. However, he mentions (and claims that his model deals with) the Bell–Kochen–Specker Theorem [1, 8], which is very similar to GHZ but requires not just locality, but non-contextuality of the hidden variables (which holds in the Kolmogorovian model).

24 E.g., both devices look at the same distant star and use photons arriving from there to determines the direction in a previously agreed way.

25 As mentioned, whenever we measure a non-commuting observable, we will have to change any hidden parameter.

26 We could also choose the following alternative (but this is not what happens in the Kolmogorovian model): We could, from the pre-determined sequence of hidden variables, define $\rho (A)$ to be the frequency for *A* as determined by the hidden variables. In that case, “approaches” will be the limit for all *A*, but the value of $\rho (A)$ will differ from quantum mechanical predictions (in those observables *A* that are not actually measured).

27 Using the “standard” notion of probability: We use independent and identically distributed random variables, each using the uniform distribution. In other words, we use the product measure of countably many copies of the Lebesgue measure on [0, 1].

28 I.e., $\lim _{n\rightarrow \infty }\frac{|\{1\le m\le n:\, r_m\in A_i\}|}{n}$ exists and is equal to $a_i$.

29 I.e., if $f_i:=\lim _{n\rightarrow \infty }\frac{|\{1\le m\le n:\, r_m\in A_i\}|}{n}$ is defined.

30 We may need some implications of the Axiom of Choice that allow us to work with the Hilbertspace in the usual way; but we do not need the partition of the reals into continuum many disjoint unmeasurable sets.
